# Mitigation of Acrylamide Content in Biscuits through Combined Physical and Chemical Strategies

**DOI:** 10.3390/foods11152343

**Published:** 2022-08-05

**Authors:** Emanuela Lo Faro, Tommaso Salerno, Giuseppe Montevecchi, Patrizia Fava

**Affiliations:** 1 Department of Life Sciences (Agri-Food Science Area), University of Modena and Reggio Emilia, Via Amendola 2 (Pad. Besta), 42124 Modena, Italy; 2 BIOGEST-SITEIA Interdepartmental Centre, University of Modena and Reggio Emilia, Piazzale Europa 1, 42124 Modena, Italy

**Keywords:** neo-formed contaminant, steam release, ammonium bicarbonate replacement, QuEChERS, acrylamide Toolbox

## Abstract

Acrylamide in biscuits represents a major concern. This research work was aimed at modifying the current formulation of biscuits to reduce the acrylamide content while maintaining the chemical, physical, and sensory characteristics of the original product. A strategy based on the FoodDrinkEurope Acrylamide Toolbox was adopted. The content of the leavening agent ammonium bicarbonate, the baking temperature program, and the time duration of steam released during the baking process were the three factors evaluated through a factorial design of experiment. The partial replacement of ammonium bicarbonate (from 9.0 g to 1.5 g per 500 g of flour) with sodium bicarbonate (from 4.5 g to 12.48 g), lowering of the temperature in the central phase of the baking process (from 170 °C to 150 °C), and the release of steam for 3 min resulted in an 87.2% reduction in acrylamide concentration compared to biscuits of reference. CIELab color indices and *a_w_* were the parameters that showed the most significant correlation with acrylamide concentration in biscuits and could, therefore, become markers to predict the acrylamide content along production lines for an instant evaluation.

## 1. Introduction

Acrylamide is a neo-formed contaminant (NFC) that has been found in different kinds of foods that contain a high quantity of carbohydrates and are cooked by baking, roasting, or frying at temperatures higher than 100 °C [1]. In 1994, acrylamide was classified by IARC as Group 2A (potential carcinogen and neurotoxic to humans). This contaminant has genotoxic properties and causes carcinogenesis since it bonds strongly with DNA [2]. For these reasons, acrylamide levels in food are strictly controlled by European Food Safety Authority (EFSA) [3].

The highest levels of contamination, among the foods investigated, have been found in french fries, coffee, and biscuits. The European authorities have established benchmark levels for food classes in Regulation (EU) n. 2017/2158 (Attachment IV). The critical acrylamide threshold for biscuits and waffles is 350 μg/kg_FW_, while remarkable attention has been put on infant biscuits, setting a very low threshold of 150 μg/kg_FW_. Because baby foods can pose significantly higher health risks due to the lower body weight of infants, baby food manufacturers have to abide by stringent parameters and undergo careful scrutiny.

Most acrylamide is formed primarily through the Maillard reaction, which specifically involves the amino group of asparagine, the carbonyl group of reducing sugars, and intermediate molecules of the Maillard reaction [4]. Another way acrylamide forms is through a reaction that involves acrolein [5]. Both chemical reactions are strongly dependent on the time–temperature factor, which means that the final result is similar when high temperatures and a short processing time or the reverse are applied [6].

Currently, there are no technological strategies to completely prevent acrylamide formation, although there are some ways to mitigate its concentration in food [7,8,9,10]. The acrylamide Toolbox lists the intervention steps that may be applied to reduce formation of acrylamide in specific manufacturing processes and products. The “ALARA” principle (As Low As Reasonably Achievable), which the Toolbox relies upon, states that the acrylamide presence needs to be reduced to the minimum, taking into account the risk presented, other legitimate considerations, such as potential risks from other contaminants, sensory properties and quality of the final product, and the feasibility and effectiveness of controls [11]. Moreover, the most effective methods applied so far have involved: (i) use of the asparaginase enzyme [12,13], (ii) use of different kinds of flours with low asparagine levels [14], (iii) replacement of ammonium bicarbonate (through the release of NH_3_, which supports the presence of Maillard reaction intermediates such as glyoxal and methylglyoxal) with non-ammoniagenic leavening agents [15,16], and (iv) assessment of different methods of baking, such as steaming (40% relative humidity), vacuum baking, and time–temperature optimization [11,17]. In fact, steam application helps to move the water activity (*a_w_*) value from the Maillard reaction’s optimum, hence takes place more slowly [11].

While reducing the quantity of acrylamide in biscuits is paramount, keeping the biscuits’ sensory characteristics, such as color and size, without losing their nutritional value is also essential. However, asparaginase poses several technological challenges for which the main objective of studies on food technologies aims to find suitable alternative strategies. These challenges include: asparaginase, which is currently and effectively employed as an acrylamide-mitigating agent [13], but makes the confectionery production process less cost-effective; the commercial preparation of the enzyme comes from the microorganism *Aspergillus niger*, a classified self-cloned micro-organism thus considered a genetically modified organism (GMO) and recently banned from the “organic” regime regulations.

Many researchers have focused their attention on the recipe modification, considering the influence of the substitution of inverted sugar with sucrose [18] and the replacement of wheat with other grains [14]. Good results have been obtained with the introduction of legume flours [19,20], even if sometimes the sensory properties are not always accepted by consumers [19]. The effect of cookie ingredients and cookie formulation has also been studied [21]. Of course, processing, i.e., the time–temperature condition of baking, plays an important role in acrylamide formation [8]. Oven-type baking processes affect acrylamide formation [18]. In particular, baking in ventilated mode promotes acrylamide formation, unlike baking in static mode [22]. Conversely, microwave baking has been studied for its capability to reduce thermal processing hazards and, consequently, acrylamide concentrations [23]. Vacuum-combined baking possesses these same capabilities and seems to be able to reduce acrylamide by about 30% in comparison with conventional baking [24].

Despite the possibly infinite combinations of recipes and baking conditions and the feasibility of such solutions in an industrial environment, this work considered only a slight modification of the recipe (i.e., the ratio of two different leavening agents) and the introduction of steam into a phase of the time–temperature program with a fixed total baking time, emulating the industrial conditions. Therefore, the present work aimed to investigate the influence, on the acrylamide amount in biscuits, of four baking conditions that differ in time/temperature cooking programs and use two different times of steam release to prepare biscuits from two recipes in which the ratios of the leavening agents ammonium bicarbonate/sodium bicarbonate were equal to 1:8 and 1:2. To achieve this goal, a factorial design of experiment (FDOE) 2^3^ was applied to produce eight batches of experimental biscuits that were compared with reference biscuits obtained from standard recipes and baking conditions. As a secondary goal, size parameters (thickness, length, and width), color coordinates in the CIELab space (L*, a*, and b*), and water activity of the sample biscuits were measured to assess the magnitude of their correlation with the acrylamide content.

## 2. Materials and Methods

### 2.1. Biscuit Preparation

**Figure 1:**
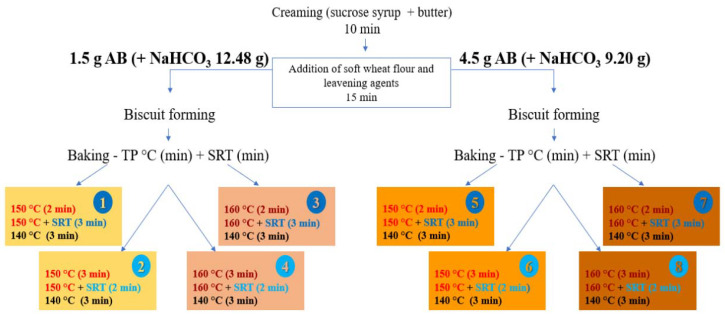
Diagram of the whole process and of the sample set obtained through the FDOE. AB, ammonium bicarbonate; TP, baking temperature program; SRT, steam release time.

The ingredients: soft wheat flour (Italian type “0”), sugar syrup, fats, flavoring agents, and leavening agents (sodium bicarbonate and ammonium bicarbonate) were bought at a local market. The formulation for the biscuits was obtained using the creaming method, which consisted of adding the ingredients into a kneading machine equipped with a leaf-shape beater (PastaMatic Gourmet 1950 Edition, De Longhi Appliances Srl, Campi Bisenzio, Italy), following two steps (Figure 1): (i) mixing the liquid ingredients (i.e., sucrose syrup and butter) for 10 min; and (ii) adding the powders (i.e., soft wheat flour and leavening agents) and thoroughly homogenizing them for 15 min.

Once the doughs were obtained, biscuits were molded using stainless steel cookie cutters according to the following dimensions: thickness 2 mm, width 2.5 mm, and length 6 mm. The samples were baked in an electric oven (EKF 616 E UD, Tecnoeka S.r.l., Borgoricco, Italy) for 8 min. For each batch, 12 biscuit doughs positioned in the same arrangement on a perforated stainless-steel pan were baked. The pan was placed in the oven chamber on the third shelf (out of six) from the top.

Preliminary tests were carried out to optimize the temperature program to emulate as closely as possible the physical and sensory characteristics of a standard industrial biscuit (SIB). A reference biscuit (RIB) was prepared using a standard recipe and without the introduction of steam during baking. Aside from the use as a control sample, one RIB batch per week (for a total of three batches) was prepared to rule out every possible influence coming from changing environmental conditions.

### 2.2. Factorial Design of Experiment (FDOE)

An FDOE was created to optimize the number of experiments. The choice fell on a 2^3^ FDOE to assess the three most critical factors: the steam release time (SRT), the baking temperature program (TP), and the amount of leavening agent (ammonium bicarbonate, AB). For each of the eight experiments, Figure 1 shows the levels of the three factors selected for the FDOE application, which were established after accurate tests on the TP to obtain experimental samples as similar as possible to the SIB. The oven temperature was controlled using the thermocouple system with an indication on the oven display and by introducing a certified thermometric probe with an accuracy of ±1 °C.

The steam was produced using tap water connected to the oven hose nozzle through a plastic pipeline. The release was regulated to achieve a relative humidity of 40% inside the oven.

Different relative quantities of leavening agents were used. For the preparation of 500 g of dough, the RIB contained ammonium bicarbonate 9.00 g and sodium bicarbonate 4.50 g, whereas the samples called “4.5 g AB” contained ammonium bicarbonate 4.50 g and sodium bicarbonate 9.20 g, and, finally, the samples called “1.5 g AB” contained ammonium bicarbonate 1.50 g and sodium bicarbonate 12.48 g (Figure 1).

The eight batches of biscuits from FDOE were cooked according to a balanced Latin square to compose an arrangement of eight rows and eight columns and considering only the first three columns as replicates (Table 1). The replicates were prepared in 3 days, once a week. The reference biscuits were also cooked on the same day.

After baking, biscuits were left to sit for 60 min until complete cooling was achieved. For each of the eight FDOE points, eight biscuits were wrapped and sealed in oriented polypropylene bags using a FoodSaver sealing bar (IFS001X, JCS Europe Ltd., Cheadle, UK) and kept until analyses that were scheduled 7 days later.

### 2.3. Chemicals

All solvents and reagents were of analytical grade. Acrylamide, with a purity of 99% to be used as the external standard, acrylamide-2,3,3-d_3_ (acrylamide d_3_), with a purity of 99% to be used as the internal standard, and acetonitrile (Chromasolv^®^ Plus purity for LC-MS) were obtained from Sigma-Aldrich Merck KGaA (Milan, Italy).

QuEChERS pouches containing MgSO_4_ 4.0 g + NaCl 0.5 g were purchased from Agilent Technologies Italia S.p.A. (Milan, Italy). Deionized water was obtained through an Elix 3^UV^ purification system (Millipore Merck KgaA, Milan, Italy).

### 2.4. Biscuit Size

The size parameters (thickness, length, and width) of the biscuits were measured 1 h after baking with the use of a vernier caliper.

### 2.5. Color Determination

Color was measured using a tristimulus colorimeter (Spectrophotometer CM-700d, Konica Minolta, Milan, Italy) in transmittance mode over the visible spectrum (from 380 to 770 nm), using the illuminant D65 and 10° standard observer [25]. The parameters of the CIELab space L* (brightness), a* (green-red axis), and b* (blue-yellow axis) were measured on all of the baked biscuits (12 per experimental condition).

Color distance (ΔE) was calculated as reported in the literature [26] using the following equation:

ΔE = [(L*_1_ − L*_2_)^2^ + (a*_1_ − a*_2_)^2^ + (b*_1_ − b*_2_)^2^]^½^ (1)

where 1 e 2 are two different samples.

Hue angle (h°) [22] was also calculated according to the following formula:

h° = [arctan (b*/a*)/2 π] × 360(2)

### 2.6. Water Activity (a_w_)

The *a_w_* was measured on three biscuits for each sample using an AQUALAB 4TE water activity meter (Decagon Devices Inc., Pullman, WA, USA).

### 2.7. Extraction Protocol for Acrylamide

For each one of the eight FDOE points, experimental biscuits (around 35 g) were finely ground with a blender. The powder obtained was separated into its particle size classes using decreasing pore size sieves (Giuliani Tecnologie S.r.l., Torino, Italy) with the following meshes: 850 μm, 500 μm, 212 μm, and 63 μm. The powder fraction recovered on the 63 μm sieve (size within 63–212 μm) was used for the extraction.

An aliquot of 2.50 g from each gross sample was transferred into a 50 mL polypropylene flat-top screw cap tube, acrylamide d3 (500 uL; 0.4 mg/kg) was added, and a ceramic homogenizer for QuEChERS was introduced. Afterwards, 7 mL of water and 10 mL of acetonitrile were introduced, and the tube was manually shaken for 1 min following the addition of each solvent. A ready-to-use mixture of QuEChERS pouch composed of MgSO_4_ 4.0 g + NaCl 0.5 g was added and manually shaken for 1 min and then vortexed for 3 min to facilitate the acrylamide migration into the acetonitrile phase. Each tube was twice centrifuged for 3 min at 3500 rpm to assure the separation of the layers. A portion of the upper layer (measured at exactly 5.0 mL) was withdrawn and evaporated to dryness using a rotational vacuum concentrator (Eppendorf concentrator 5301, Eppendorf, Hamburg, Germany). The sample was re-dissolved in 1 mL of water. Following centrifugation (Mini Spin, Eppendorf Italy, Milan, Italy), the supernatant of each sample was introduced into a 2 mL vial. The same extraction procedure was carried out and analyzed in triplicate using three different aliquots of each ground gross sample.

### 2.8. LC-ESI-MS/MS Optimized Analytical Conditions

Acrylamide determination was carried out through liquid chromatography coupled with mass spectrometry using an Agilent Technologies RP-LC-ESI-MS/MS triple quad system (Santa Clara, CA, USA), consisting of an HPLC (Agilent Technologies 1200 Series) equipped with a degasser, a binary pump, an autosampler, and a 6410B triple quadrupole mass spectrometer.

The MS/MS parameters (parent ion, product ion, fragmentor, collision energy, and polarity of the ESI ion source) were optimized using standard solutions (0.125 mg/L) of acrylamide and acrylamide d3 through their individual introduction into the MS source. Selected reaction monitoring (SRM) transitions of the most abundant fragments (product ions) were used.

Vials containing the samples were then loaded into an autosampler carousel at a controlled temperature (20 °C), and 5 μL of each sample were injected into a Gemini RP C_18_ column (Phenomenex, Torrance, CA, USA) (25 cm × 2 mm i.d. × 5 μm particle size × 110 Å pore size). The solvent system was composed of water with formic acid 0.1% (solvent A) andacetonitrile with formic acid 0.1% (solvent B). The elution (17 min of run + 10 min of post-run) was carried out according to the following gradient: 0% B (6.5 min), 95% B (7.5 min), 95% B (13.0 min), 0% B (13.1 min), 0% B (17.0 min) with a flow rate of 0.23 mL/min at a controlled temperature of 20 °C (pressure of about 180 bars at run start).

Source parameters were optimized as follows: gas (N_2_) temperature, 300 °C; N_2_ flow, 3 L/min; nebulizer pressure, 30 psi; capillary, positive 2500 V and negative 1500 V. Peak identification included comparison of peak retention times to those obtained with calibrants and evaluation of the tandem mass spectroscopic experiments. Quantification was performed by external standard calibration in the presence of acrylamide d3 as the internal standard, and the chromatograms were processed using MassHunter Workstation Quantitative Analysis software vB05.00 (Agilent Technologies Inc., Santa Clara, CA, USA).

### 2.9. Evaluation of Linearity, Limit of Detection and Lower Limit of Quantification (Detectability), Intra-Day and Inter-Day Repeatability (Precision), and Recovery Test (Trueness)

Solutions with increasing concentrations of diluted pure standards were prepared to evaluate the linearity of the instrumental response. The range of concentrations (0.02–1.00 mg/L) was established on the basis of the data already present in the literature and by exploring the chromatographic traces of some real samples. Because biscuits can cause a matrix effect that can affect acrylamide determination, linearity was also evaluated using an analyte-free biscuit matrix in which known concentrations of acrylamide were added.

In the aqueous reference solution, the instrumental limit of detection (LOD) and the lower limit of quantification (LLOQ) were obtained by applying the equation:

LOD or LLOQ = (K × sy/x)/b(3)

where sy/x and b are the estimated regression standard deviation and the slope of the relevant analytical calibration function, respectively. K = 3 and K = 10 were chosen in order to obtain the LOD and LLOQ, respectively.

Precision was evaluated with an intra-day repeatability test on a sample and a standard solution of acrylamide (0.125 mg/L) each injected five times, and an inter-day repeatability test carried out on a sample injected over the course of five consecutive days in the same conditions. The relative standard deviations were calculated for each substance.

The trueness was evaluated through a recovery test where a known quantity of a standard acrylamide solution (0.25 mg/L) was added in the presence of the internal standard in samples consisting of a biscuit matrix previously deprived of the analyte and applying the extraction and chromatographic determination protocols.

### 2.10. Statistical Analysis

Employing different aliquots of each sample, three repetitions of each measurement were performed for each kind of analytical determination. Data were expressed as mean values (±standard deviations). FDOE was set using Microsoft Excel 365.

Because parametric ANOVA assumption tests (such as normality, equal variance and equal or near-equal sample size) were not satisfied, we performed the non-parametric Kruskal–Wallis equality-of-population rank test [27] and the non-parametric Wilcoxon rank-sum (Mann–Whitney U test) [28], using the temperature program (TP), the steam release time (SRT), and the ammonium bicarbonate percentage (AB) as statistical factors to observe the differences in median values in the sample set. Nevertheless, the ANOVA was also run, and its results were compared to those of the Kruskal–Wallis test.

An evaluation of the significant correlation among all parameters that showed significant differences in the Wilcoxon rank-sum (Mann–Whitney U test), i.e., thickness, L*, a*, b*, *a_w_*, and acrylamide concentration, was performed using the non-parametric Spearman rank order correlation test to assess correlations among acrylamide and the other variables. All tests were performed using Stata/SE 11.0 for Windows (StataCorp LP, College Station, TX, USA).

## 3. Results and Discussion

### 3.1. Optimization of the Reference Industrial Biscuit (RIB)

The use of industrial-scale machinery for the preparation of the SIB implies substantial differences compared with the samples obtained using laboratory-scale equipment. For this reason, preliminary tests were carried out to try to balance the most evident differences and obtain experimental samples with specific physical characteristics, such as thickness, *a_w_*, and color similar to those of the SIB.

The challenge of this research work lay in obtaining good reproducibility of the industrial process conditions in producing the RIB with lab equipment while simultaneously trying to emulate the characteristics of the SIB. Therefore, the first step was to analyze the differences between the two systems in detail to understand the causes of divergence between the RIB and the SIB. Some technological differences were, however, deemed insurmountable. One such difference was the ability to develop the same extent of gluten network during the creaming phase owing to the higher quantity of industrial heat that commonly triggers a more relevant leavening effect in the biscuits. Other technological differences, such as molding and the speed of production, were considered less relevant.

**Figure 2:**
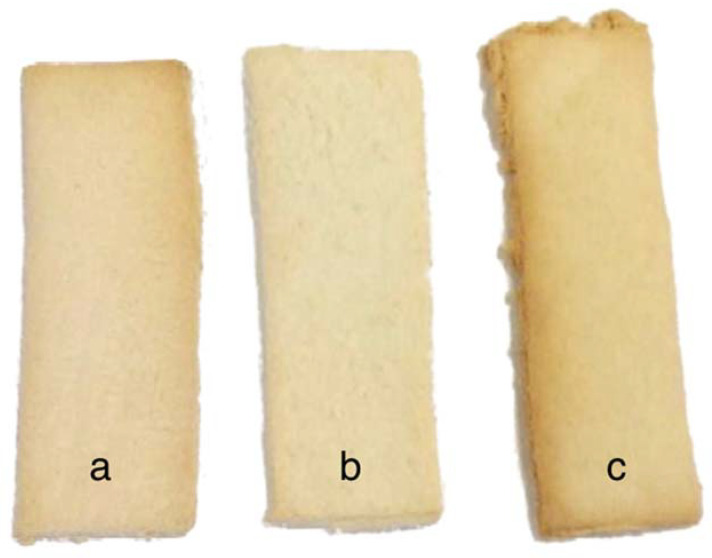
Experimental samples 4 (**a**) and 6 (**b**) and reference biscuit (RIB) (**c**).

The differences between the two baking systems may have a tremendous impact on the experimental biscuits. After several attempts, a temperature program that made RIB almost identical to the SIB was finally found (Figure 2):

160 °C (2 min) − 170 °C (3 min) − 140 °C (3 min).

However, the resulting biscuits had *a_w_* lower than that of the SIB and a slightly lower height, whereas the color was similar. Unfortunately, further variations of the TP led to a further negative impact on one of the measured parameters (*a_w_*), although height and color were very close to the original results. For this reason, it was decided to keep the height and color of the RIB as similar as possible to the SIB, to the detriment of the *a_w_*.

### 3.2. Steam Release and Temperature Program Assessment

Attempts to include the steam release in the FDOE led to further fine-tuning of the baking TP. Furthermore, the steam released during baking in the FDOE would certainly have increased *a_w_* values of the samples (becoming closer to the SIB value).

Once the optimal 3-phase TP was set up, it was decided that the steam would be released in the middle phase rather than the initial or final phases for two different reasons: First, the initial phase was excluded because the most intense chemical leavening of the product takes place during this phase, and water supply would have considerably altered this phenomenon. Second, it was not sensible to introduce steam in the final phase when the biscuits needed to lose water to reach proper dryness. Had this recommendation been disregarded, a product with a higher *a_w_* would have been obtained, reducing its shelf-life. Additionally, the biscuit would have been sensorially altered, reducing its friability and, therefore, its consumer acceptability.

Following the introduction of steam, four different TP around the optimal one, each consisting of three phases, were included in the FDOE:

150 °C (2 min) − 150 °C (3 min + STR 40% r.h.) − 140 °C (3 min)

150 °C (3 min) − 150 °C (2 min + STR 40% r.h.) − 140 °C (3 min)

160 °C (2 min) − 160 °C (3 min + STR 40% r.h.) − 140 °C (3 min)

160 °C (3 min) − 160 °C (2 min + STR 40% r.h.) − 140 °C (3 min)

### 3.3. Validation of the MS/MS Parameters

Table 2 shows the optimized parameters (dwell time, fragmentor voltage, and collision energy). Both the coefficients of determination (R^2^) of the acrylamide to acrylamide d3 ratio calibration straight lines that were calculated using (1) aqueous acrylamide solutions of known concentration and (2) analyte-free biscuit matrix with additions of known acrylamide concentrations were higher than 0.99 (Table 2). This demonstrated a satisfactory linear correlation between concentration and response, also confirmed by visual inspection [29].

Regarding detectability, the LOD and LLOQ values were respectively 0.03 mg/L and 0.10 mg/L, which are far lower than the concentration of acrylamide in the extracts and also lower compared with the values found in previous studies carried out with SPE extraction [30,31]. Precision was well within 10% in all tests performed.

Trueness was assessed in terms of percentage recovery of acrylamide solutions spiked at different concentrations using biscuit matrix previously deprived of the analyte. Results showed an excellent average recovery of 98.31%.

### 3.4. Comparison between the Acrylamide Concentrations of the Eight FDOE Samples and the Reference Biscuits (RIB)

Table 3 shows the results for the acrylamide concentrations obtained in the FDOE sample set as well as in the RIB samples. All of the samples belonging to the FDOE showed acrylamide concentrations lower than those of the RIB, demonstrating that the combination of using a lower baking temperature with the introduction of steam in the middle step along with decreasing the amount of ammonium bicarbonate in the recipe is a promising strategy to mitigate acrylamide in biscuits. The Kruskal–Wallis equality-of-population rank test was run to highlight any significant difference among the sample medians, in particular those belonging to the FDOE (samples 1 to 8) and the RIB. This non-parametric test showed a moderately significant difference across the samples (*p* = 0.0530). Although nonapplicable, the one-way ANOVA also showed significant differences (*p* ≤ 0.001) among the samples. Among the FDOE samples, a post hoc test showed significant differences only for samples 1 and 8, which exhibited the lowest and the highest acrylamide concentrations, respectively. The comparison of the conditions applied to the preparation of samples 1 and 8 indicated that all experimental factors (SRT, TP, and AB) were substantially different (Figure 1). The former showed an 87.2% reduction in acrylamide in comparison with the RIB, whereas the latter showed a 63.2% reduction.

In sample 1, the TP used and the amount of AB in the recipe were the lowest. At the same time, the amount of steam administered was the maximum in terms of time. As hypothesized, this combination of parameters resulted in the lowest acrylamide value among all of the samples, whereas the diametrically opposite conditions of TP, RST, and AB were applied to sample 8, in which the highest amount of acrylamide was found within the FDOE.

Samples 2 and 5 followed sample 1 in terms of acrylamide content. These samples were obtained with the lowest baking TP and only one of the other two factors most favorable to the mitigation of acrylamide (1.5 g of AB and 3 min of SRT for samples 2 and 5, respectively). Sample 3 also had only one factor out of three that differed from sample 1, namely the highest baking TP. However, TP caused the most intense effect on acrylamide formation, as widely demonstrated [8,9,10] and recommended [11]. It emerged that sample 3 showed contaminant concentrations higher than those in samples 2 and 5. Finally, samples 4, 6, and 7, all of which had higher acrylamide values than the previous samples, showed only one favorable mitigation factor out of three, i.e., the lowest AB amount, the lowest TP, and the highest SRT, respectively.

The obtained data seemed to demonstrate the formulated hypothesis, according to abundant scientific evidence derived from experimental works on biscuits and concerning the use of steam-cooking [32] or on different starchy matrices, such as bread and potatoes [10,32,33]. As far as the contribution to acrylamide formation by AB is concerned, the present data agree with data from the scientific literature and demonstrate that diminishing this leavening agent is an interesting approach: some authors [21] concluded that ammonium bicarbonate is the most effective ingredient in terms of causing the formation of acrylamide in biscuits and that its complete elimination from the recipe may be a solution. However, the complete replacement of a leavening agent such as AB would require the reformulation of the industrial process to obtain a biscuit appreciated by consumers.

### 3.5. All Parameters Measured on the Eight FDOE Samples

The application of the Wilcoxon rank-sum (Mann–Whitney U test) made it possible to reach some general considerations on the contributions that the individual three factors made to the formation of acrylamide in the experimental biscuit matrix. For this reason, the RIB was excluded from this test. Table 4 shows that a significant reduction in the acrylamide concentration in the experimental biscuits was obtained by decreasing the amount of AB in the recipe, using 1.5 g instead of 4.5 g, while also reducing the baking TP (150 °C in the initial phase and in the middle phase instead of 160 °C in both of them). However, as for the two SRT (2 and 3 min), no significant effect was shown with respect to acrylamide concentration.

The concentration of acrylamide increased in parallel with the increase in both the TP and AB due to the crucial role that these two factors play in triggering and quickening the Maillard reaction and the non-enzymic browning in general, while SRT seemed to play a less relevant role.

The statistical analysis was also extended to the other parameters analyzed. In addition to acrylamide concentration in biscuits, Table 3 shows the data concerning the values of the sample sizes, the CIELab color coordinates, and *a_w_*. The Wilcoxon rank-sum (Mann–Whitney U test) was applied to evaluate the influence of TP, SRT, and AB on the studied parameters (Table 4). As for the size parameters, length and width did not show any significant effect, whereas SRT significantly affected thickness (*p* = 0.0574). Indeed, by enhancing the SRT, the biscuit thickness significantly increased.

The small differences deriving from the manual preparation of the forms did not cause any substantial changes in length and width during the baking process. Conversely, the SRT influenced the thickness of the samples. This phenomenon could be explained by making different hypotheses: (i) the steam flow could have reduced the water loss from the biscuit because it decreases the vapor pressure between the surrounding environment and the biscuit surface [33], (ii) when the water evaporates, it stretches the gluten net where it is physically trapped, since, when the water tries to leave the dough, the latter is dragged upwards due to the passage of the water [33].

Contrarily, TP and AB did not have any significant effect on biscuit thickness. Temperature variation did not affect the rate of decomposition of the leavening agents, which occurs at temperatures far lower than 100 °C [34]. Concerning the AB concentration, the results show that the partial replacement of this leavening agent with sodium bicarbonate-cream of tartar was successful, and it did not cause any changes in the structure of the biscuits [15].

Regarding the color parameters, the wide difference in the RIB values compared with the experimental samples of the FDOE was clear. In the RIB samples, the values of redness (a*) and yellowness (b*) increased consistently with the increase in acrylamide concentration, to the detriment of values of brightness (L*), which were at the same time reduced.

Having excluded RIB from the statistical comparison to carry out a finer evaluation of the combination of the FDOE factors, other aspects also emerged. L* was significantly affected (*p* = 0.0016) by AB level, causing higher brightness when AB increased. This influence was also revealed by other authors [23,35], also considering different elaborations of the CIELab color parameters: for example, the E-value [36] and the browning index (BI) [22], both dependent on the L* values. The a* coordinate showed a significant effect for TP (*p* = 0.0238), giving a higher tendency to redness as temperature was increased. A weaker effect was shown by a* due to AB (*p* = 0.0659), while AB heavily affected the b* coordinate (*p* = 0.0008), thus giving a more intense yellowness as higher temperatures and lower AB levels were applied.

The higher concentration of AB significantly increased L* and decreased b* in the FDOE samples. This twofold effect was related to the ammonia release [11]. Contrarily, the lower concentration of AB entailed a higher concentration of sodium ions (from sodium bicarbonate), which yielded a darker biscuit surface color during baking [37,38].

On the other hand, the effect of TP on the behavior of color parameters was more predictable. Higher TP values significantly affected a* and b*, increasing both values. Taking into consideration that a* refers to the value on the green–red axis and b* refers to the value on the blue–yellow axis, it can be assumed that the more intense the thermal damage produced, the more evident the browning phenomenon on the surface.

Another way to compare colors of different samples is the evaluation of the color distance (ΔE). ΔE is a dimensionless parameter that arises from the combination of the L*, a*, and b* values when pairs of samples are considered. ΔE of sample pairs leads to determining whether or not there is a difference in the colors perceived by the human eye according to specific thresholds [39], i.e., ΔE < 0.2 indicates an imperceptible difference between colors; 0.2 < ΔE < 0.5, a very small difference between colors; 0.5 < ΔE < 1.5, a small difference between colors; 2 < ΔE < 3, a barely distinguishable difference between colors; 3 < ΔE < 6, a very distinguishable difference between colors; 6 < ΔE < 12, a large color difference; and ΔE >12, completely different colors.

Table 5 shows the data obtained when applying these thresholds to the ΔE of sample pairs in the present work. This diversity of colors could easily be distinguished by the naked eye for most of the FDOE samples when compared to the RIB, while with regards to samples 5 and 6, the “difference” was even larger. In order to further investigate this point, specific sensory analyses should be run to understand if this discrepancy may lead consumers to reject the product.

The last parameter considered was *a_w_*. The different levels of TP and AB did not have significant effects on the *a_w_* in biscuits, while this *a_w_* was affected only by SRT (Table 4). Indeed, samples 1, 3, 5, and 7, obtained with a 3 min RST, showed higher *a_w_* values than those obtained with a 2 min application only. For this reason, a careful assessment of shelf-life in combination with the product’s sensory properties should be taken into consideration. However, *a_w_* values of the FDOE biscuits were around 0.3, which represents a reference value for this type of food matrix.

### 3.6. Study of the Correlations among Parameters

At the end of the study, an evaluation of the significant correlations was carried out to establish if one or more of the other parameters could be considered as chemical markers of the quantity of acrylamide present in the samples. This evaluation included all of the biscuits produced, those originating from both the FDOE and the RIB samples. One of the most interesting correlations was found between acrylamide and *a_w_* (r = −0.73; *p* ≤ 0.001). It was a negative correlation, meaning the higher the *a_w,_* the lower the acrylamide content. Acrylamide was also positively correlated to the thickness to the *a_w_* ratio (r = 0.66; *p* ≤ 0.01).

Furthermore, acrylamide was positively correlated with a* (r = 0.57; *p* ≤ 0.02). However, the most relevant correlation was found with a combination of the three CIELab color coordinates, that is, h° (r = −0.84; *p* ≤ 0.001); (a*/b*) × L (r = 0.87; *p* ≤ 0.001); and a*/b* (r = 0.84; *p* ≤ 0.001), as some authors have previously described [22,40]. This would lead to the prediction of the extent of acrylamide content in samples by comparing the coupling of *a_w_* and CIELab color indices. The lower the *a_w_* and the higher the color indices, the higher the acrylamide concentration in biscuits.

## 4. Conclusions

The present research study showed that proper modulation of the baking temperature, as well as partial replacement of the ammonium bicarbonate in the recipe with a more suitable leavening agent, are the physical and chemical factors that can be jointly applied to mitigate the formation of acrylamide in biscuits. The steam release during the central baking phase favorably affected the thickness and water content, parameters critically linked to consumers’ acceptability of the biscuits.

With the best combination of the factors explored, an 87.2% average reduction in acrylamide content was achieved compared with the reference sample, which was produced in the laboratory to mimic the industrial biscuit.

The significant correlations of the acrylamide content with the *a_w_* and CIELab color indices are compelling, as variables of this type could easily be evaluated by means of an optical sensor placed on the production line to exploit the properties of NIR spectroscopy and determine the color CIELab parameters by reflectance. Furthermore, the possibility of gathering a larger database of data could likely lead to highlighting other relevant relationships between color indices and acrylamide content.

Finally, this work lays the foundations for further in-depth investigations, where different mitigation techniques could be combined with those of the present study with the goal of lowering the acrylamide content in other types of food products.

## Figures and Tables

**Table 1:** Latin square design used to randomize the cooking of the eight batches of biscuits, according to the FDOE design.

Batches from FDOE	Rep. 1	Rep. 2	Rep. 3
1	*1*	*2*	*8*
2	*2*	*3*	*1*
3	*3*	*4*	*2*
4	*4*	*5*	*3*
5	*5*	*6*	*4*
6	*6*	*7*	*5*
7	*7*	*8*	*6*
8	*8*	*1*	*7*

**Table 2:** List of the main MS parameters optimized and results of the method validation.

Analyte	ESI		MW (g/mol)	Parent Ions (*m/z*)	Product Ions (*m/z*)	Dwell Times (msec)	Fragmentor Voltage (V)	Collision Energy (V)		
Acrylamide	+		71.08	72.1 (M + H)^+^	55	80	70	8		
Acrylamide d3	+		74.10	75.1 (M + H)^+^	58	80	70	8		
	**Linearity**			**Detectability**		**Trueness**		**Precision**		
	**Range of** **concentration** **(mg/L)**	**R^2^** **(aqueous solutions of acrylamide of known concentration)**	**R^2^** **(analyte-free biscuit matrix with addition of known acrylamide concentrations)**	**LOD** **(mg/L)**	**LLOQ** **(mg/L)**	**% recovery**		**Intra-day on a biscuit sample (*n* = 5)** **(RDS %)**	**Intra-day using a standard solution of acrylamide (*n* = 5)** **(RDS %)**	**Inter-day on a biscuit sample (*n* = 5)** **(RDS %)**
Acrylamide	0.02 ÷ 1.00	0.995	0.998	0.03	0.10	98.31		5.57	2.61	6.16

ESI: electrospray ionization; R^2^: coefficient if determination; LOD: limit of detection; LLOQ: lower limit of quantification (LLOQ); RSD, relative standard deviation.

**Table 3:** Results of measurements of size, CIELab color parameters, water activity, and acrylamide (expressed as mg/kg_FM_; _FM_: fresh matter) carried out on FDOE samples (samples 1–8) and reference samples (RIB).

Samples	Thickness (mm)	Length (mm)	Width (mm)	L*	a*	b*	*a_w_*	Acrylamide (mg/kg_FM_)	Percentage Reduction Compared with RIB (%)
**1**	5.7 ± 0.5	80.7 ± 1.4	27.6 ± 2.2	75.41 ± 0.91	6.36 ± 0.62	30.53 ± 0.68	0.325 ± 0.018	0.229 ± 0.010	87.2
**2**	5.6 ± 0.5	80.4 ± 1.1	26.5 ± 1.6	75.81 ± 0.89	6.25 ± 0.47	30.67 ± 0.60	0.271 ± 0.034	0.363 ± 0.005	79.7
**3**	5.7 ± 0.6	80.4 ± 1.7	26.6 ± 1.6	74.39 ± 1.17	7.27 ± 0.63	31.35 ± 0.62	0.298 ± 0.026	0.401 ± 0.007	77.6
**4**	5.6 ± 0.7	80.0 ± 1.2	27.4 ± 1.6	74.61 ± 1.07	7.00 ± 0.69	31.21 ± 0.74	0.244 ± 0.035	0.455 ± 0.003	74.6
**5**	6.0 ± 0.8	80.6 ± 1.4	27.6 ± 2.0	76.94 ± 1.03	5.76 ± 0.53	27.69 ± 0.85	0.384 ± 0.039	0.372 ± 0.004	79.2
**6**	5.3 ± 0.4	79.8 ± 1.7	27.2 ± 1.8	77.25 ± 1.00	5.91 ± 0.73	27.91 ± 1.03	0.257 ± 0.036	0.470 ± 0.030	73.7
**7**	6.1 ± 0.4	79.0 ± 1.3	26.8± 1.4	76.62 ± 0.97	6.36 ± 0.64	28.60 ± 0.80	0.337 ± 0.029	0.478 ± 0.010	73.3
**8**	5.8 ± 0.6	80.0 ± 1.8	26.7 ± 1.6	76.60 ± 0.94	6.65 ± 0.74	29.04 ± 1.09	0.245 ± 0.053	0.658 ± 0.092	63.2
**RIB**	5.9 ± 0.6	79.1 ± 1.3	27.9 ± 1.6	71.71 ± 2.26	9.64 ± 1.09	33.10 ± 1.11	0.109 ± 0.031	1.788 ± 0.111	

The colors used to indicate the samples and cell backgrounds refer to the colors used in Figure 1.

**Table 4:** Wilcoxon rank-sum (Mann–Whitney U test).

	Thickness (mm)	Length (mm)	Width (mm)	L*	a*	b*	*a_w_*	Acrylamide (mg/kg_FM_)
**TP**	n.s.	n.s.	n.s.	n.s.	0.0238	n.s.	n.s.	0.0356
**SRT**	*0.0574*	n.s.	n.s.	n.s.	n.s.	n.s.	**0.0022**	n.s.
**AB**	n.s.	n.s.	n.s.	**0.0016**	*0.0659*	**0.0008**	n.s.	0.0312

Results of the Wilcoxon rank-sum (Mann–Whitney U test) in the samples set are reported as *p*-value. *p* < 0.001, very strong significant effect (bold and underlined); *p* < 0.01, strong significant effect (bold); *p* < 0.05, moderate significant effect (standard); *p* < 0.1, weak significant effect (italics); *p* > 0.1, n.s.: not significant. SRT: steam release time; TP: temperature program; AB: ammonium bicarbonate percentage.

**Table 5:** Color distance (ΔE) calculated for all possible pairs of samples. Samples 1–8 belong to the FDOE; RIB is the reference biscuit.

	1	2	3	4	5	6	7	8	RIB
**1**									
**2**	0.43								
**3**	1.87	1.59							
**4**	1.51	1.23	0.37						
**5**	3.23	3.29	4.41	4.71					
**6**	3.13	3.23	4.36	4.67	0.42				
**7**	2.23	2.28	3.36	3.66	1.03	1.14			
**8**	1.86	1.93	2.97	3.25	1.50	1.66	0.53		
**RIB**	3.82	3.99	3.60	3.62	6.70	6.89	5.46	5.90	

## Data Availability

The data are available from the corresponding author.
